# Correction to: Machine learning-based reconstruction of 2D MRI for quantitative morphometry in epilepsy

**DOI:** 10.1162/IMAG.x.1280

**Published:** 2026-06-11

**Authors:** Corey Ratcliffe, Peter N. Taylor, Christophe de Bézenac, Kumar Das, Shubhabrata Biswas, Anthony Marson, Simon S. Keller

**Affiliations:** Clinical Imaging Sciences Centre, School of Psychology, University of Sussex, Falmer, United Kingdom; Department of Neuro Imaging and Interventional Radiology, National Institute of Mental Health and Neuro Sciences, Bangalore, India; CNNP Lab, Interdisciplinary Computing and Complex BioSystems Group, School of Computing, Newcastle University, Newcastle upon Tyne, United Kingdom; Department of Pharmacology and Therapeutics, Institute of Systems, Molecular, and Integrative Biology, University of Liverpool, Liverpool, United Kingdom; The Walton Centre NHS Foundation Trust, Liverpool, United Kingdom

In the original article, we report a clinical cohort comprised of healthy controls (HC), and two phenotypic subgroups of people with idiopathic generalised epilepsy: 42 people with drug-resistant idiopathic generalised epilepsy, and 33 (31 following exclusions) people with drug-sensitive idiopathic generalised epilepsy—pwDRIGE and pwDSIGE in the original manuscript, respectively. Following a review of archived data, it was flagged that these phenotypic labels had been mistakenly assigned at an early stage in this study. The pwDRIGE subgroup was instead comprised of 42 people with drug-resistant focal epilepsy, and the pwDSIGE group 31 people with IGE (11 of whom were drug-sensitive). Group definitions have been changed to reflect this, but importantly, subgroup characteristics remain unchanged and are consistent with the original manuscript. The updated demographic information is available in Table 1 (a revision of Table 2 from the original manuscript) and Table 2 (a revision of Table S1 from the original Supplementary Material). An update to Supplementary Figure 7 shows the relabelled results of subgroup comparisons (see Fig. 1).

**Fig. 1.**
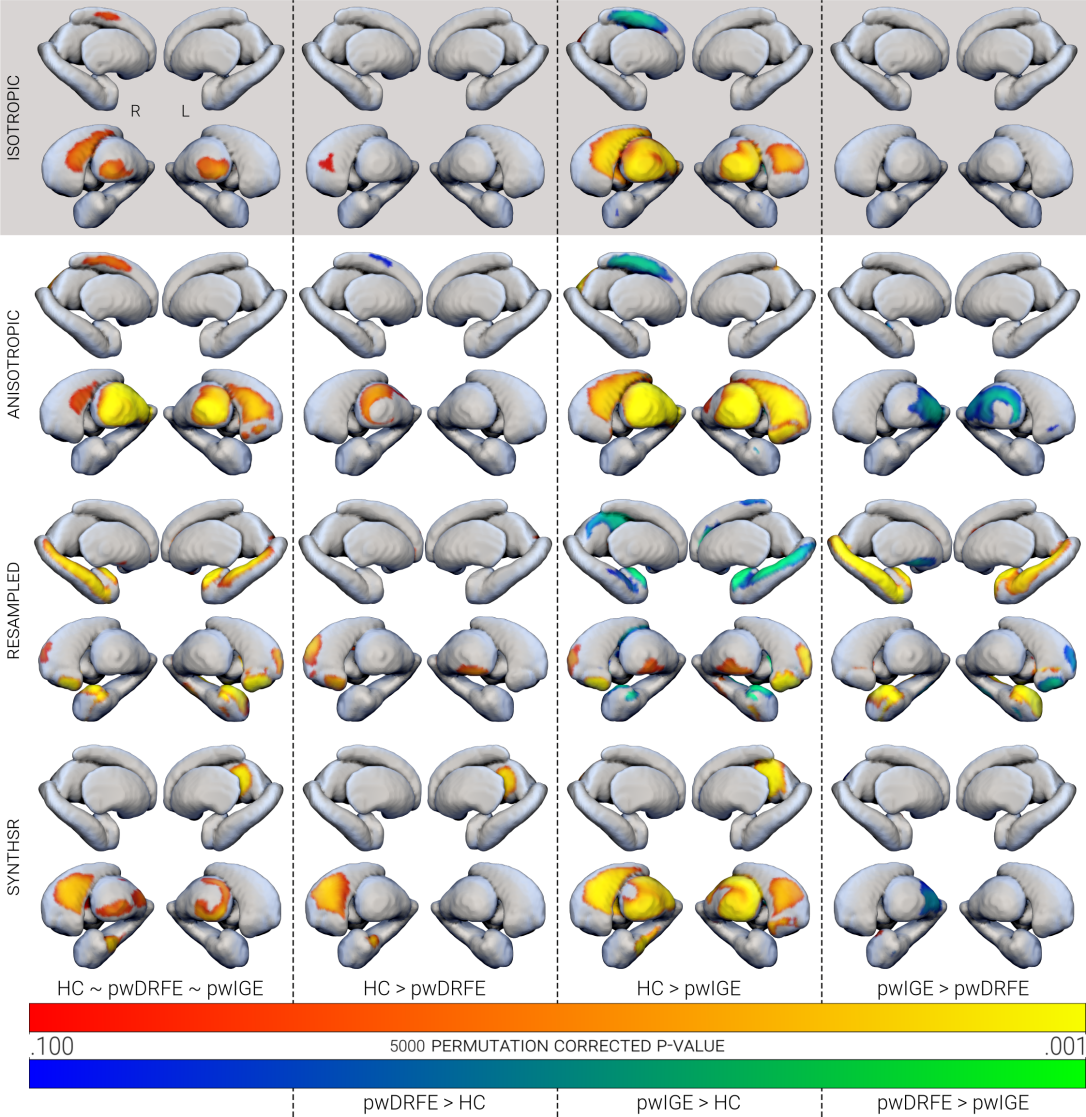
Clusters of subcortical surface shape deformations between people with drug-resistant focal epilepsy, people with idiopathic generalised epilepsy, and healthy controls, observed across image types. Only clusters with *p corr* <.100 are shown, which were derived with one-sided permutation (*n-perm =* 5000) testing. The direction of the contrast is presented next to the relevant colourbar. HC, Healthy Controls; pwDRFE, people with drug-resistant focal epilepsy; pwIGE, people with Idiopathic Generalised Epilepsy.

**Table 1.** Revised demographics.

	Healthy controls		People with drug-resistant focal epilepsy		People with idiopathic generalized epilepsy			
	(*n = 39*)		(*n = 42*)		(*n = 31*)		*p =*	
Age at scan in years mean (*SD*)	32.38	(*8.65*)	31.76	(*11.12*)	32.16	(*14.20*)	f-test:	.969
							HC/pwDRFE:	.805
							HC/pwIGE:	.935
							pwIGE/pwDRFE:	.882
TPV in mm^3^ mean (*SD*)	1583688	(*136239*)	1558160	(*176741*)	1594837	(*159805*)	f-test:	.593
							HC/pwDRFE:	.472
							HC/pwIGE:	.771
							pwIGE/pwDRFE:	.332
Sex F/M (*F%*)	23/16	(*58.67%*)	25/17	(*59.52%*)	17/14	(*54.84%*)	f-test:	.913
							HC/pwDRFE:	.960
							HC/pwIGE:	.728
							pwIGE/pwDRFE:	.690

Sample demographics from the different groups within the cohort. For age at scan and TPV, differences in group variance were assessed with ANOVAs and uncorrected post-hoc pairwise t-tests. Differences in sex distributions were assessed with group and pairwise chi-square tests. TPV was calculated from the isotropic images, using FreeSurfer’s recon-all command. HC; Healthy Controls; pwDRFE, People with Drug-resistant Focal Epilepsy; pwIGE, people with Idiopathic Generalised Epilepsy; TPV, Total Parenchymal Volume.

**Table 2.** Revised sample demographics.

Study ID	Sex	Age at scan	Group	Total parenchymal volume (mm^3^)	Study ID	Sex	Age at scan	Group	Total parenchymal volume (mm^3^)
sub-001	F	34	pwIGE	1500325.74	sub-057	F	41	HC	1549914.99
sub-002	F	23	pwIGE	1542291.40	sub-058	F	19	pwDRFE	1695651.18
sub-003	M	19	pwIGE	1861860.90	sub-059	M	19	pwDRFE	1734680.68
sub-004	F	19	pwIGE	1396310.05	sub-060	F	35	pwDRFE	1301169.69
sub-005	M	25	pwIGE	1637994.16	sub-061	F	21	HC	1472779.06
sub-006	F	60	pwIGE	1493748.84	sub-062	M	52	pwDRFE	1499155.29
sub-007	M	24	pwIGE	1549106.18	sub-063	F	33	HC	1497035.28
sub-008	F	21	pwIGE	1587915.52	sub-064	F	32	HC	1431230.78
sub-009	F	32	pwIGE	1588225.40	sub-065	F	34	HC	1454569.74
sub-010	M	38	pwIGE	1511318.51	sub-066	F	30	pwDRFE	1680187.58
sub-011	M	67	pwIGE	1533209.66	sub-067	M	37	HC	1927143.97
sub-012	F	46	pwIGE	1495030.25	sub-068	M	35	HC	1562383.85
sub-013	M	20	pwIGE	1953398.17	sub-069	F	45	HC	1466323.57
sub-014	F	24	pwIGE	1460892.16	sub-070	F	21	pwDRFE	1369512.90
sub-015	M	35	pwIGE	1653925.05	sub-071	M	26	HC	1713088.98
sub-016	M	18	pwIGE	1564495.70	sub-072	F	34	pwDRFE	1490116.37
sub-017	M	39	pwIGE	1423657.57	sub-073	M	38	HC	1722978.46
sub-018	F	24	pwIGE	1995978.41	sub-074	F	27	HC	1531661.85
sub-019	M	21	pwIGE	1726997.75	sub-075	F	34	HC	1452227.41
sub-020	F	36	pwIGE	1755597.02	sub-076	F	29	pwDRFE	1428725.09
sub-021	F	31	pwIGE	1522907.27	sub-077	M	43	HC	1658457.47
sub-022	F	31	pwIGE	1529185.05	sub-078	F	43	pwDRFE	1259660.17
sub-023	M	23	pwIGE	1586201.15	sub-079	M	19	pwDRFE	1804608.90
sub-024	F	19	pwIGE	1740328.94	sub-080	M	25	HC	1824748.21
sub-025	F	58	pwIGE	1473819.61	sub-081	F	45	pwDRFE	1408431.19
sub-026	F	18	pwIGE	1405915.12	sub-082	M	38	HC	1740562.68
sub-027	M	22	pwIGE	1816224.09	sub-083	F	23	pwDRFE	1505731.72
sub-028	M	24	pwIGE	1656447.61	sub-084	M	38	pwDRFE	1783313.46
sub-029	F	56	pwIGE	1690206.23	sub-085	F	27	pwDRFE	1356596.93
sub-030	F	57	pwIGE	1391252.34	sub-086	F	36	pwDRFE	1562572.83
sub-031	M	33	pwIGE	1395181.14	sub-087	M	39	pwDRFE	1496065.78
sub-032	M	22	HC	1756746.92	sub-088	F	39	HC	1550714.93
sub-033	M	23	HC	1632795.68	sub-089	M	22	pwDRFE	1538654.83
sub-034	M	23	HC	1628394.95	sub-090	F	25	pwDRFE	1733437.92
sub-035	F	61	pwDRFE	1559574.71	sub-091	M	18	pwDRFE	1916703.41
sub-036	M	22	HC	1700479.91	sub-092	F	26	HC	1570408.41
sub-037	M	28	pwDRFE	1686863.59	sub-093	F	28	pwDRFE	1412396.84
sub-038	F	27	HC	1724797.87	sub-094	M	29	pwDRFE	1932541.28
sub-039	M	37	HC	1696736.56	sub-095	F	28	HC	1491109.73
sub-040	M	22	pwDRFE	1506507.38	sub-096	F	32	HC	1397883.20
sub-041	F	25	HC	1443795.57	sub-097	M	39	HC	1718302.02
sub-042	F	60	HC	1433847.43	sub-098	F	27	HC	1599759.26
sub-043	M	22	pwDRFE	1799045.87	sub-099	M	34	HC	1768257.64
sub-044	F	18	pwDRFE	1404680.91	sub-100	F	25	HC	1545723.81
sub-045	F	27	pwDRFE	1646535.98	sub-101	F	24	HC	1456575.91
sub-046	F	26	HC	1587856.12	sub-102	M	29	pwDRFE	1559425.30
sub-047	F	41	pwDRFE	1555237.06	sub-103	F	50	pwDRFE	1402154.62
sub-048	F	50	HC	1454538.28	sub-104	F	40	pwDRFE	1396666.03
sub-049	M	41	pwDRFE	1741355.94	sub-105	M	35	pwDRFE	1513924.64
sub-050	F	54	pwDRFE	1326614.89	sub-106	F	24	HC	1629950.58
sub-051	M	32	HC	1413972.33	sub-107	F	19	pwDRFE	1429645.26
sub-052	F	39	pwDRFE	1582578.70	sub-108	F	22	pwDRFE	1341604.36
sub-053	M	47	pwDRFE	1876719.97	sub-109	F	21	pwDRFE	1520121.37
sub-054	F	38	HC	1722298.96	sub-110	M	22	pwDRFE	1683436.33
sub-055	F	38	pwDRFE	1326222.36	sub-111	M	43	HC	1480935.45
sub-056	F	28	HC	1352827.48	sub-112	M	27	pwDRFE	1673906.88

Total sample demographics. F, Female; HC, Healthy Controls; M, Male; pwDRFE, people with Drug-resistant Focal Epilepsy; pwIGE, people with Idiopathic Generalised Epilepsy.

This error does not impact on our primary conclusions, which regarded the reliability of image processing methods and was agnostic to the demographics of the included participants. Furthermore, the groupings used in our secondary clinical analyses remain the same. Nonetheless, we interpreted several of our findings in the context of a homogenous idiopathic generalised epilepsy cohort, whereby systematic differences could be reasonably attributed to drug resistance—see section 4.2. of the original manuscript. These interpretations are no longer wholly valid, and we therefore offer an update to the clinical summary from section 4.2.

As stated in the original article, data is available on request from the authors, now with corrected metadata. Additionally, on request from the authors, a version of the original manuscript is available where all errors have been highligted. Code (unchanged) freely available on GitHub: https://github.com/C-Ratcliffe/221216_Proj-IS.

## 4.2 Revised Clinical Summary

With modern analysis techniques, imaging abnormalities in pwE are reported with increasing frequency and evidence suggests that associated structural abnormalities extend beyond putative atrophy, potentially including abnormal orientations or regional subcortical thickening (hypertrophy) (Caciagli et al., 2019; Zubal et al., 2025). Limited by a heterogenous epilepsy sample of a modest size, and the relative heterogeneity of the epilepsy phenotype, we opted to focus on examining subcortical morphometry for the clinical arm of this project. (McKavanagh et al., 2023; Whelan et al., 2018) We also present clinical interpretations tentatively.

A subset of the data analysed in this paper has been examined previously by Brownhill et al. (2021), who found evidence of pallidal, putaminal, and thalamic volumetric reductions (relative to controls) in the truncated IGE-only cohort. IGE is a heterogenous subtype of epilepsy, the phenotypes of which the present sample is insufficient to represent reliably—especially when combined into a mixed-aetiology pwE group. Nonetheless, our clinical analyses indicate convergent structural abnormalities in individuals with IGE and FE, consistent with the concept of a core epileptogenic network. This study therefore recapitulates and expands on the findings of previous structural imaging analyses into the neural correlates of epilepsy.

Firstly by extending the findings of Brownhill et al. (2021) and identifying bilateral pallidal volume reductions in a mixed-aetiology epilepsy cohort, but also by demonstrating that whilst volumetric analyses might lack the necessary sensitivity to identify pathology-specific abnormalities in other key regions, subcortical shape analyses offer a means to identify pathology-related focal atrophy and hypertrophy. Specifically, we highlight abnormal inwards surface deflation in the accumbens, caudate, and thalamus (concordant with volumetric abnormalities found in Whelan et al. (2018)), and outwards regional inflation in the caudate-despite the lack of any volumetric abnormalities, such as those specific to the pallidum.

Despite the inclusion of a focal epilepsy subgroup, hypertrophy of the amygdalae was not present (and was not indicated in supplementary subgroup analyses). Whilst commonly associated with mTLE, there is no consistent evidence suggesting relating increased amygdalae volumes to IGE, suggesting instead some potential influence on the distinct cognitive profiles of mTLE and IGE (Ratcliffe et al., 2020; Zubal et al., 2025). Our findings support the hypothesis that the interaction between structural abnormalities and phenotype in epilepsy are multifaceted and complex, and that further focused study could deliver insights into the impact of subcortical/projection network reorganisation on seizure presentation and comorbid cognitive impairment (Rodriguez-Cruces et al., 2022).
