# Supplementary material for: Machine learning-based reconstruction of 2D MRI for quantitative morphometry in epilepsy
Source: Imaging Neurosci (Camb). 2025 Nov 10;3:IMAG.a.997. doi: 10.1162/IMAG.a.997 (PMC12603658; doi:10.1162/IMAG.a.997)
Supplement: Supplementary Material [file IMAG.a.997_supp.pdf]

Machine Learning-based Reconstruction of 2D MRI for Quantitative Morphometry in Epilepsy

Corey Ratcliffe,[1,2*] Peter N. Taylor,[1] Christophe de Bézenac,[3] Kumar Das,[4] Shubhabrata Biswas,[4] Anthony Marson,[3,4] Simon S. Keller[3,4]

[1]*CNNP Lab, Interdisciplinary Computing and Complex BioSystems Group, School of Computing, Newcastle University, Newcastle upon Tyne, UK*

[2]*Department of Neuro Imaging and Interventional Radiology, National Institute of Mental Health and Neuro Sciences, Bangalore, India*

[3]*Department of Pharmacology and Therapeutics, Institute of Systems, Molecular, and Integrative Biology, University of Liverpool, Liverpool, UK*

[4]*The Walton Centre NHS Foundation Trust, Liverpool, UK*

[*]*Correspondence: corey.ratcliffe@newcastle.ac.uk*

October 15, 2025

A Supplementary Materials

Supplementary Material S1

Table s1. Sample demographics

Study ID	Sex	Age at Scan	Group	Total Parenchymal Volume (mm^3)	Study ID	Sex	Age at Scan	Group	Total Parenchymal Volume (mm^3)
sub-001	F	34	pwDSIGE	1500325.74	sub-057	F	41	HC	1549914.99
sub-002	F	23	pwDSIGE	1542291.40	sub-058	F	19	pwDRIGE	1695651.18
sub-003	M	19	pwDSIGE	1861860.90	sub-059	M	19	pwDRIGE	1734680.68
sub-004	F	19	pwDSIGE	1396310.05	sub-060	F	35	pwDRIGE	1301169.69
sub-005	M	25	pwDSIGE	1637994.16	sub-061	F	21	HC	1472779.06
sub-006	F	60	pwDSIGE	1493748.84	sub-062	M	52	pwDRIGE	1499155.29
sub-007	M	24	pwDSIGE	1549106.18	sub-063	F	33	HC	1497035.28
sub-008	F	21	pwDSIGE	1587915.52	sub-064	F	32	HC	1431230.78
sub-009	F	32	pwDSIGE	1588225.40	sub-065	F	34	HC	1454569.74
sub-010	M	38	pwDSIGE	1511318.51	sub-066	F	30	pwDRIGE	1680187.58
sub-011	M	67	pwDSIGE	1533209.66	sub-067	M	37	HC	1927143.97
sub-012	F	46	pwDSIGE	1495030.25	sub-068	M	35	HC	1562383.85
sub-013	M	20	pwDSIGE	1953398.17	sub-069	F	45	HC	1466323.57
sub-014	F	24	pwDSIGE	1460892.16	sub-070	F	21	pwDRIGE	1369512.90
sub-015	M	35	pwDSIGE	1653925.05	sub-071	M	26	HC	1713088.98
sub-016	M	18	pwDSIGE	1564495.70	sub-072	F	34	pwDRIGE	1490116.37
sub-017	M	39	pwDSIGE	1423657.57	sub-073	M	38	HC	1722978.46
sub-018	F	24	pwDSIGE	1995978.41	sub-074	F	27	HC	1531661.85
sub-019	M	21	pwDSIGE	1726997.75	sub-075	F	34	HC	1452227.41
sub-020	F	36	pwDSIGE	1755597.02	sub-076	F	29	pwDRIGE	1428725.09
sub-021	F	31	pwDSIGE	1522907.27	sub-077	M	43	HC	1658457.47
sub-022	F	31	pwDSIGE	1529185.05	sub-078	F	43	pwDRIGE	1259660.17
sub-023	M	23	pwDSIGE	1586201.15	sub-079	M	19	pwDRIGE	1804608.90
sub-024	F	19	pwDSIGE	1740328.94	sub-080	M	25	HC	1824748.21
sub-025	F	58	pwDSIGE	1473819.61	sub-081	F	45	pwDRIGE	1408431.19
sub-026	F	18	pwDSIGE	1405915.12	sub-082	M	38	HC	1740562.68
sub-027	M	22	pwDSIGE	1816224.09	sub-083	F	23	pwDRIGE	1505731.72
sub-028	M	24	pwDSIGE	1656447.61	sub-084	M	38	pwDRIGE	1783313.46
sub-029	F	56	pwDSIGE	1690206.23	sub-085	F	27	pwDRIGE	1356596.93
sub-030	F	57	pwDSIGE	1391252.34	sub-086	F	36	pwDRIGE	1562572.83
sub-031	M	33	pwDSIGE	1395181.14	sub-087	M	39	pwDRIGE	1496065.78
sub-032	M	22	HC	1756746.92	sub-088	F	39	HC	1550714.93
sub-033	M	23	HC	1632795.68	sub-089	M	22	pwDRIGE	1538654.83
sub-034	M	23	HC	1628394.95	sub-090	F	25	pwDRIGE	1733437.92
sub-035	F	61	pwDRIGE	1559574.71	sub-091	M	18	pwDRIGE	1916703.41
sub-036	M	22	HC	1700479.91	sub-092	F	26	HC	1570408.41
sub-037	M	28	pwDRIGE	1686863.59	sub-093	F	28	pwDRIGE	1412396.84
sub-038	F	27	HC	1724797.87	sub-094	M	29	pwDRIGE	1932541.28
sub-039	M	37	HC	1696736.56	sub-095	F	28	HC	1491109.73
sub-040	M	22	pwDRIGE	1506507.38	sub-096	F	32	HC	1397883.20
sub-041	F	25	HC	1443795.57	sub-097	M	39	HC	1718302.02
sub-042	F	60	HC	1433847.43	sub-098	F	27	HC	1599759.26
sub-043	M	22	pwDRIGE	1799045.87	sub-099	M	34	HC	1768257.64
sub-044	F	18	pwDRIGE	1404680.91	sub-100	F	25	HC	1545723.81
sub-045	F	27	pwDRIGE	1646535.98	sub-101	F	24	HC	1456575.91
sub-046	F	26	HC	1587856.12	sub-102	M	29	pwDRIGE	1559425.30
sub-047	F	41	pwDRIGE	1555237.06	sub-103	F	50	pwDRIGE	1402154.62
sub-048	F	50	HC	1454538.28	sub-104	F	40	pwDRIGE	1396666.03
sub-049	M	41	pwDRIGE	1741355.94	sub-105	M	35	pwDRIGE	1513924.64
sub-050	F	54	pwDRIGE	1326614.89	sub-106	F	24	HC	1629950.58
sub-051	M	32	HC	1413972.33	sub-107	F	19	pwDRIGE	1429645.26
sub-052	F	39	pwDRIGE	1582578.70	sub-108	F	22	pwDRIGE	1341604.36
sub-053	M	47	pwDRIGE	1876719.97	sub-109	F	21	pwDRIGE	1520121.37
sub-054	F	38	HC	1722298.96	sub-110	M	22	pwDRIGE	1683436.33
sub-055	F	38	pwDRIGE	1326222.36	sub-111	M	43	HC	1480935.45
sub-056	F	28	HC	1352827.48	sub-112	M	27	pwDRIGE	1673906.88

Total sample demographics. F, Female; HC, Healthy Controls; M, Male; pwDRIGE, people with Drug-resistant idiopathic generalised Epilepsy; pwDRIGE, people with drug-sensitive Idiopathic Generalised Epilepsy

Supplementary Material S2

Table s2. Wholebrain descriptive summary statistics

	Wholebrain Similarity (DSC)	Volume (z-score)	Volume (ICC)
**recon-all**			
Anisotropic	0.94	-0.13	0.97
Isotropic		Reference	
Resampled	0.94	-0.13	0.97
SynthSR	0.94	0.32	0.98
**recon-all-clinical**			
Anisotropic	0.91	-0.81	0.98
Isotropic	0.93	-0.89	0.99
Resampled	0.91	-0.79	0.99
**DL+DiReCT**			
Resampled	0.84	-2.72	0.93
Isotropic	0.86	-2.40	0.93
SynthSR	0.84	-2.52	0.96

Structural measurements for different image types, extracted from FreeSurfer `recon-all`, FreeSurfer `recon-all-clinical`, and DeepSCAN parcellations. The isotropic scans, anisotropic scans, and resampled images were processed with both FreeSurfer pipelines, the SynthSR-derived images were processed with `recon-all` only, and DeepSCAN was applied to the resampled images as part of the DL+DiReCT pipeline. Z-scores were computed relative to the distribution from the isotropic data, and ICCs, DSCs, and z-scores were computed based on wholebrain segmentation masks. DSC, Dice Similarity Coefficient; ICC, Intraclass Correlation Coefficient

Table s3. Subcortical volume comparisons

	Accumbens		Amygdala		Caudate		Hippocampus		Pallidum		Putamen		Thalamus	
	L	R	L	R	L	R	L	R	L	R	L	R	L	R
**f-test**														
Anisotropic	.123	.151	.333	.575	.817	.562	.852	.202	.243	.432	.469	.599	.224	.097
Isotropic	.781	.804	.634	.327	.655	.505	.368	.178	.140	.122	.408	.424	.383	.185
Resampled	.149	.174	**.001**	**.039**	.570	.189	**.012**	**.024**	**.028**	.052	.614	.161	.266	.211
SynthSR	**.043**	.695	.687	.510	.656	.412	.491	**.027**	.397	.905	.215	.487	.059	.365
**Healthy controls vs. people with drug-resistant IGE**														
Anisotropic	.623	.133	.330	.422	.630	.354	.940	.098	.119	.671	.234	.450	.345	.102
Isotropic	.647	.600	.865	.152	.333	.228	.709	.171	.089	.061	.354	.408	.359	.143
Resampled	.063	.099	.070	.159	.577	.277	.292	.294	**.017**	**.022**	.999	.080	.199	.183
SynthSR	**.014**	.447	.387	.314	.444	.199	.241	**.009**	.210	.806	.199	.340	**.041**	.349
**Healthy controls vs. people with drug-sensitive IGE**														
Anisotropic	.053	.085	.666	.312	.566	.359	.692	.864	.208	.172	.391	.347	.085	**.046**
Isotropic	.515	.535	.411	.295	.679	.624	.279	.649	.088	.104	.204	.200	.172	.095
Resampled	.554	.972	**.033**	.235	.366	.701	**.037**	.059	.408	.266	.453	.906	.781	.911
SynthSR	.093	.953	.920	.953	.460	.595	.777	.104	.976	.777	.098	.318	**.041**	.156
**People with drug-resistant IGE vs. people with drug-sensitive IGE**														
Anisotropic	.111	.496	.144	.929	.832	.938	.562	.177	.746	.441	.850	.772	.390	.554
Isotropic	.729	.978	.396	.763	.755	.616	.182	.084	.929	.996	.623	.596	.647	.738
Resampled	.201	.128	**.001**	**.012**	.422	.054	**.004**	**.009**	**.043**	.148	.326	.141	.144	.094
SynthSR	.659	.492	.558	.334	.663	.435	.448	.321	.297	.684	.631	.592	.850	.667

The uncorrected *p-values* from Kruskal-Wallis rank sum tests of subcortical volume differences (as measured using the FSL command, `run_first_all`) between groups, observed across image types. **Bold** values and red cells highlight statistical significance at α = .050. Healthy Controls, *n* = 39; People with drug-resistant IGE, *n* = 42; People with drug-sensitive IGE, *n* = 31. IGE, Idiopathic Generalised Epilepsy

Table s4. Subcortical surface shape comparisons

	Accumbens		Amygdala		Caudate		Hippocampus		Pallidum		Putamen		Thalamus	
	L	R	L	R	L	R	L	R	L	R	L	R	L	R
**f-test**														
Anisotropic	**.031**	.128	.731	.097	**.014**	.053	.082	.374	.359	.060	.285	.598	**.005**	**.001**
Isotropic	.213	.665	.545	.444	.057	**.047**	.231	.216	.190	.052	.372	.241	**.034**	.056
Resampled	**.001**	**.005**	**.001**	**.002**	**.009**	.054	**.002**	**.007**	**.039**	.223	.122	**.048**	.079	.157
SynthSR	.110	.099	.678	.259	.126	**.025**	.440	**.015**	.646	.076	.281	.394	**.005**	.057
**Regional inflation in Healthy controls relative to people with drug-resistant IGE**														
Anisotropic	.326	.142	.587	.956	.335	.459	.464	.863	.515	.315	.272	.248	.325	**.040**
Isotropic	.103	.263	.683	.991	.315	.093	.252	.285	.395	.293	.529	.152	.147	.142
Resampled	.093	**.029**	.211	.166	.596	**.023**	.063	.165	.142	.212	.740	**.037**	**.048**	.154
SynthSR	.147	.162	.383	.396	.245	**.024**	.418	**.023**	.511	.077	.454	.142	**.011**	.193
**Regional inflation in Healthy controls relative to people with drug-sensitive IGE**														
Anisotropic	**.005**	**.031**	.249	.194	**.004**	**.014**	.106	.192	.127	**.030**	.099	.175	**.001**	**.001**
Isotropic	.085	.560	.144	.260	**.029**	**.011**	.329	.289	.062	**.007**	.102	**.049**	**.005**	**.008**
Resampled	**.001**	**.001**	**.025**	**.023**	**.012**	**.038**	.056	.232	.073	.057	.181	.227	**.022**	.067
SynthSR	**.017**	.284	.277	.111	**.027**	**.006**	.088	**.005**	.252	**.016**	.071	.140	**.001**	**.009**
**Regional inflation in people with drug-sensitive IGE relative to people with drug-resistant IGE**														
Anisotropic	.380	.828	.434	.313	.267	.199	.360	.151	.222	.766	.810	.906	.719	.375
Isotropic	.752	.881	.424	.393	.130	.350	.357	.102	.749	.790	.966	.966	.894	.775
Resampled	**.001**	**.025**	**.001**	**.001**	.056	.189	**.001**	**.001**	**.004**	.054	.123	**.018**	.366	.346
SynthSR	.716	.861	.977	.063	.305	.714	.781	.105	.446	.343	.998	.904	.882	.432
**Regional inflation in people with drug-resistant IGE relative to healthy controls**														
Anisotropic	.525	.487	.932	.294	.119	.084	.388	.901	.815	.587	.989	.737	.815	.329
Isotropic	.061	.209	.320	.201	.399	.055	.365	.984	.997	.673	.962	.279	.905	.901
Resampled	.598	.400	.524	.606	.103	.151	.356	.593	.276	.946	.640	.232	.650	.256
SynthSR	.945	**.030**	.646	.723	.449	.067	.928	.959	.992	.125	.872	.517	.829	.291
**Regional inflation in people with drug-sensitive IGE relative to healthy controls**														
Anisotropic	.050	.912	.220	.397	**.005**	**.017**	**.017**	.147	.566	.373	.914	.945	.946	.100
Isotropic	.144	.561	.273	.110	**.010**	**.017**	.064	.092	.924	.247	.992	.644	.916	.600
Resampled	**.001**	**.001**	**.001**	**.003**	**.001**	.058	**.001**	**.016**	.206	.218	**.034**	**.043**	.345	**.023**
SynthSR	.639	.194	.608	.247	.021	**.044**	.269	.313	.589	.064	.999	.982	.449	.491
**Regional inflation in people with drug-resistant IGE relative to people with drug-sensitive IGE**														
Anisotropic	.080	.232	.704	**.019**	.151	.179	.855	.396	.144	**.022**	.245	.629	**.021**	**.025**
Isotropic	.512	.641	.408	.177	.231	.328	.599	.680	.290	.251	.432	.175	.165	.321
Resampled	**.009**	.236	**.031**	**.008**	**.042**	.515	**.017**	**.019**	**.011**	.330	.098	**.027**	.612	.479
SynthSR	.615	.793	.884	.066	.400	.631	.842	.193	.484	.313	.176	.306	.220	**.046**

Minimum *p-values* for one-sided permutation (*n-perm* = 5000) testing of subcortical surface shape deformation clusters (as measured using the FSL pipeline, `fsl_anat`) between groups, observed across image types. **Bold** values and red cells highlight statistical significance at α = .050. Healthy Controls, n = 39; People with drug-resistant IGE, n = 42; People with drug-sensitive IGE, n = 31. IGE, Idiopathic Generalised Epilepsy

Supplementary Material S3

The results of a supplementary comparison are provided below, describing the performance of DL+DiReCT depending on the type of input file provided: isotropic, resampled (as is presented in the body of this manuscript) or synthesised with SynthSR. Across all metrics, estimates were more accurate (i.e. higher ICC, higher DSC, smaller z-score differences) for the isotropic input data, performing similarly to the FreeSurfer's `recon-all-clincial` pipeline.

The combined use of the two machine learning-based models evidenced a greater susceptibility to grey matter underestimation and outlier values, although outputs were still largely comparable to the estimates from the resampled data. From this, we can infer that DL+DiReCT's attempt to analyse re-sampled data (i.e. tissue class segmentation with DeepScan) is no less accurate than SynthSR's attempted generation of an isotropic analogue—at least, for the purpose of cortical morphometry. Importantly, neither model-based approach was able to correctly impute the data that was otherwise 'missing' in the anisotropic images.

Table s5. Regional descriptive summary stats

	Cortical Regions					Subcortical Regions		
	Similarity (DSC)	Volume (z-score)	Volume (ICC)	Thickness (z-score)	Thickness (ICC)	Similarity (DSC)	Volume (z-score)	Volume (ICC)
**recon-all**								
Isotropic				Reference				
**DL+DiReCT**								
Resampled	0.63	-0.64	0.61	-2.20	0.38	0.80	-0.40	0.83
Isotropic	0.71	0.14	0.72	-0.43	0.55	0.81	-0.36	0.91
SynthSR	0.59	-0.57	0.56	-2.66	0.28	0.80	-0.42	0.79

Structural measurements for different image types, extracted from DL+DiReCT parcellations of different input images: isotropic scans, anisotropic (resampled) scans, and SynthSR-derived images. Z-scores were computed relative to the distribution from the isotropic data, and ICCs, DSCs, and z-scores were averaged over 148 cortical and 26 subcortical regions. DSC, Dice Similarity Coefficient; ICC, Intraclass Correlation Coefficient

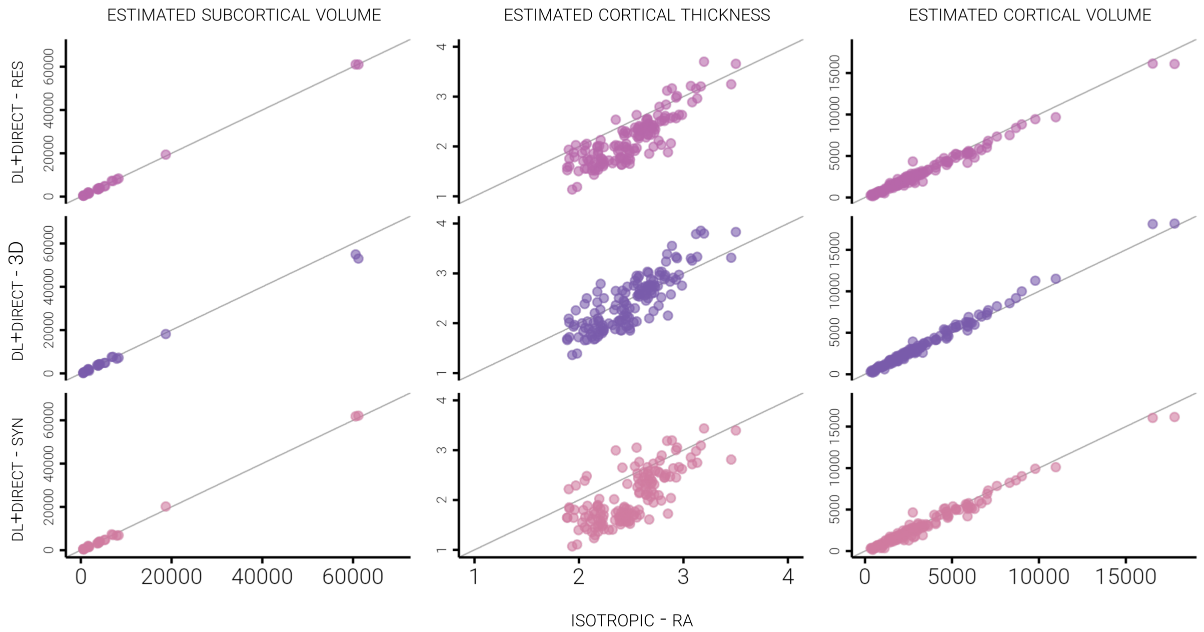

Figure s1. Scatterplots representing the relationships between FreeSurfer-extracted volume and thickness measurements of 148 parcellated cortical regions and 26 subcortical regions in the 3D-T1 scans (x-axes) and the three variable input DL+DiReCT output images (y-axes). A reference line for perfect agreement (i.e. y = x) is also plotted, demonstrating that whilst the volume estimates are relatively comparable between input files, DL+DiReCT typically underestimated the thickness values for the resampled and synthetic input data. 3D, native isotropic input; res, resampled anisotropic input; syn, SynthSR-derived input

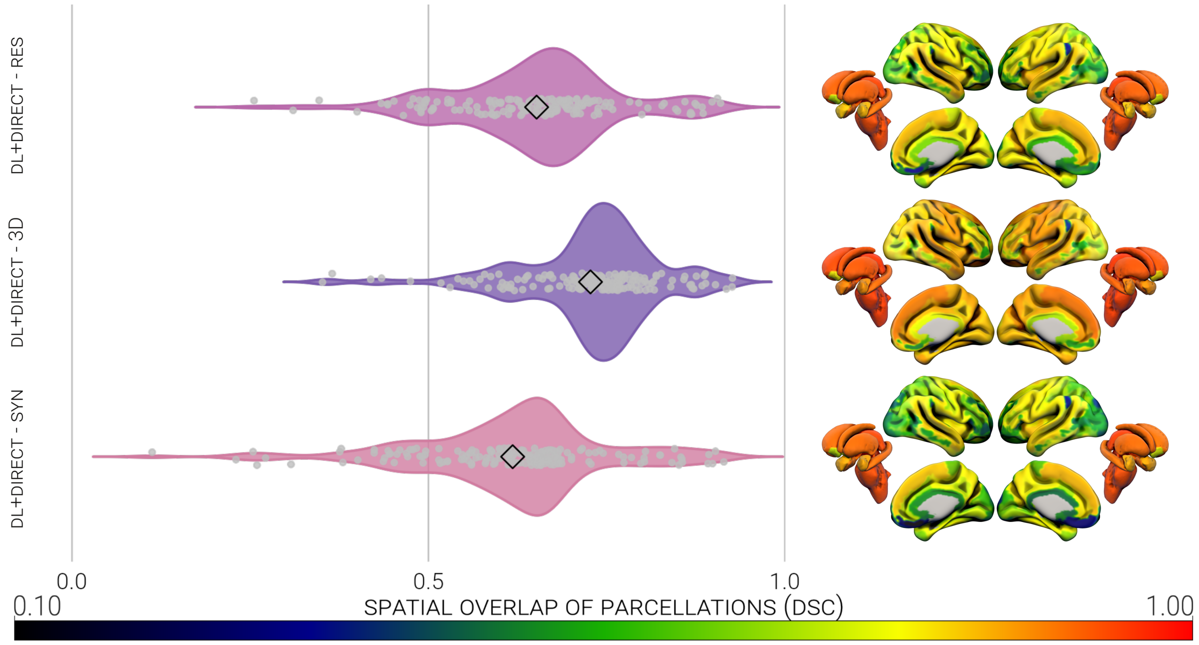

Figure s2. Heatmap and violin plot representations of the spatial overlap (presented on the scale of 0.1 to 1.0) of 174 parcellated regions created from 3D-T1 scans and analogues created using the DL+DiReCT pipeline with variable input volumes. Regional data were averaged across all participants in each image type, to leave one datum per region per image type. DSC, Dice Similarity Coefficient; 3D, native isotropic input; res, resampled anisotropic input; syn, SynthSR-derived input

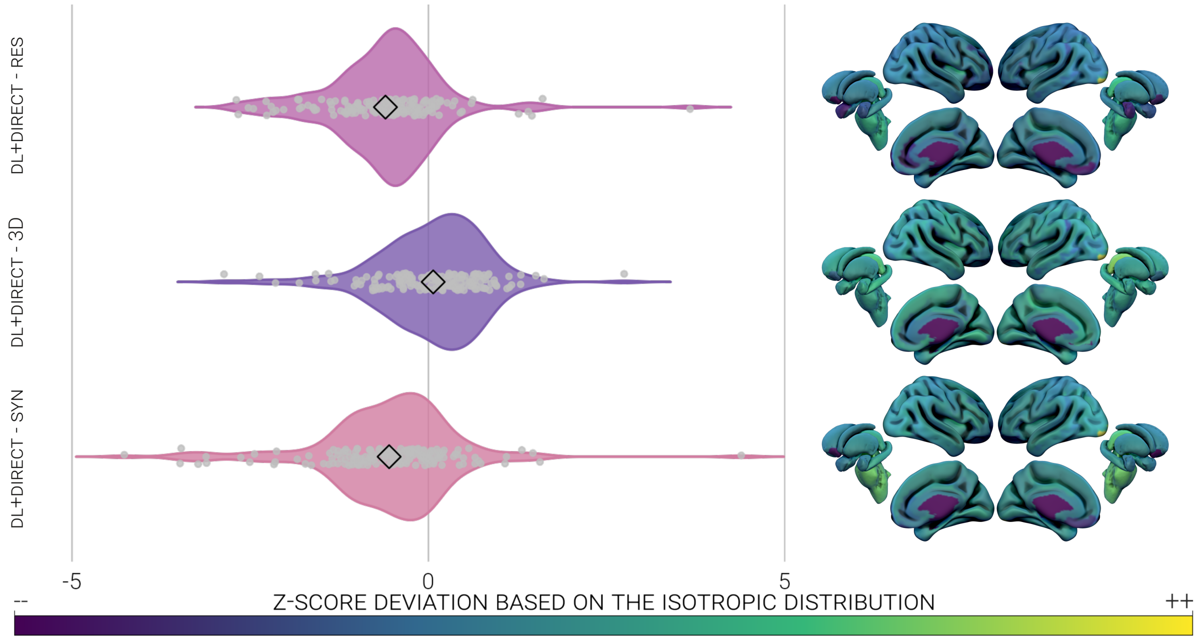

Figure s3. Heatmap and violin plot representations of the volume estimation variance (presented on the scale of -5 to +5 SD) of 174 parcellated regions created using the DL+DiReCT pipeline with variable input volumes. Regional volume estimates were first standardised to the distribution of the same region in the isotropic data. Regional data were averaged across all participants in each image type, to leave one datum per region per analogue. For heatmap creation, the values were then normalised to between 0 and 1 for each image type - these heatmaps therefore represent relative distributions of estimation bias, with the scale being bounded to the minimum and maximum z-scores for that image type. 3D, native isotropic input; res, resampled anisotropic input; syn, SynthSR-derived input

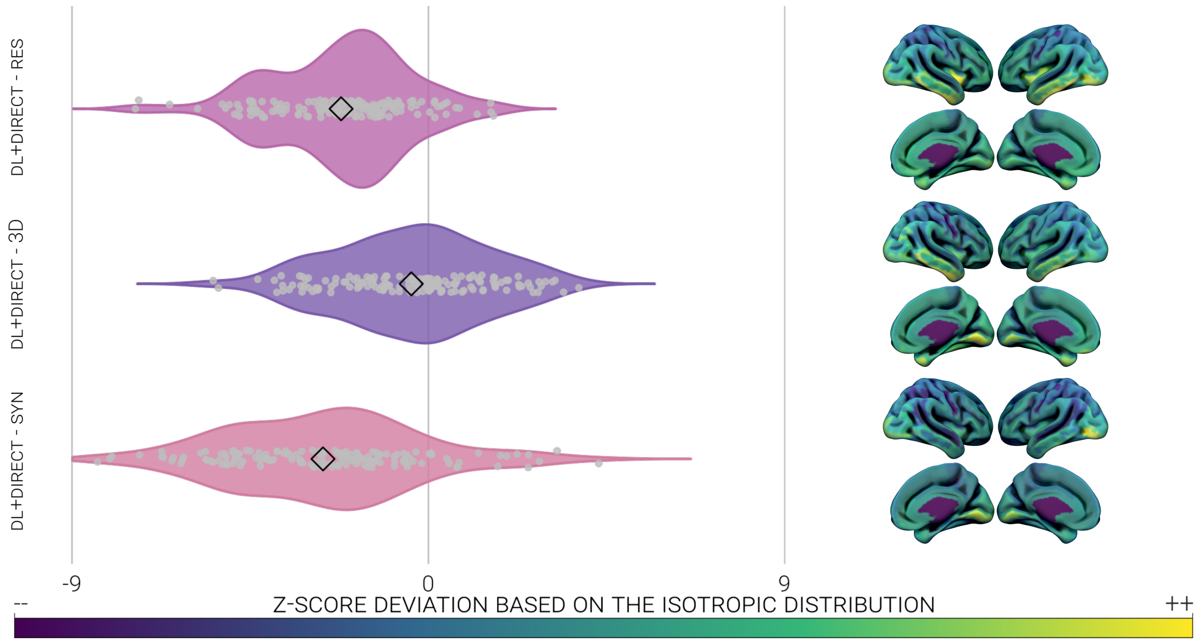

Figure s4. Heatmap and violin plot representations of the thickness estimation variance (presented on the scale of -9 to +9 SD) of 148 parcellated regions created using the DL+DiReCT pipeline with variable input volumes. Regional thickness estimates were first standardised to the distribution of the same region in the isotropic data. Regional data were averaged across all participants in each image type, to leave one datum per region per analogue. For heatmap creation, the values were then normalised to between 0 and 1 for each image type - these heatmaps therefore represent relative distributions of estimation bias, with the scale being bounded to the minimum and maximum z-scores for that image type. 3D, native isotropic input; res, resampled anisotropic input; syn, SynthSR-derived input

Supplementary Material S4

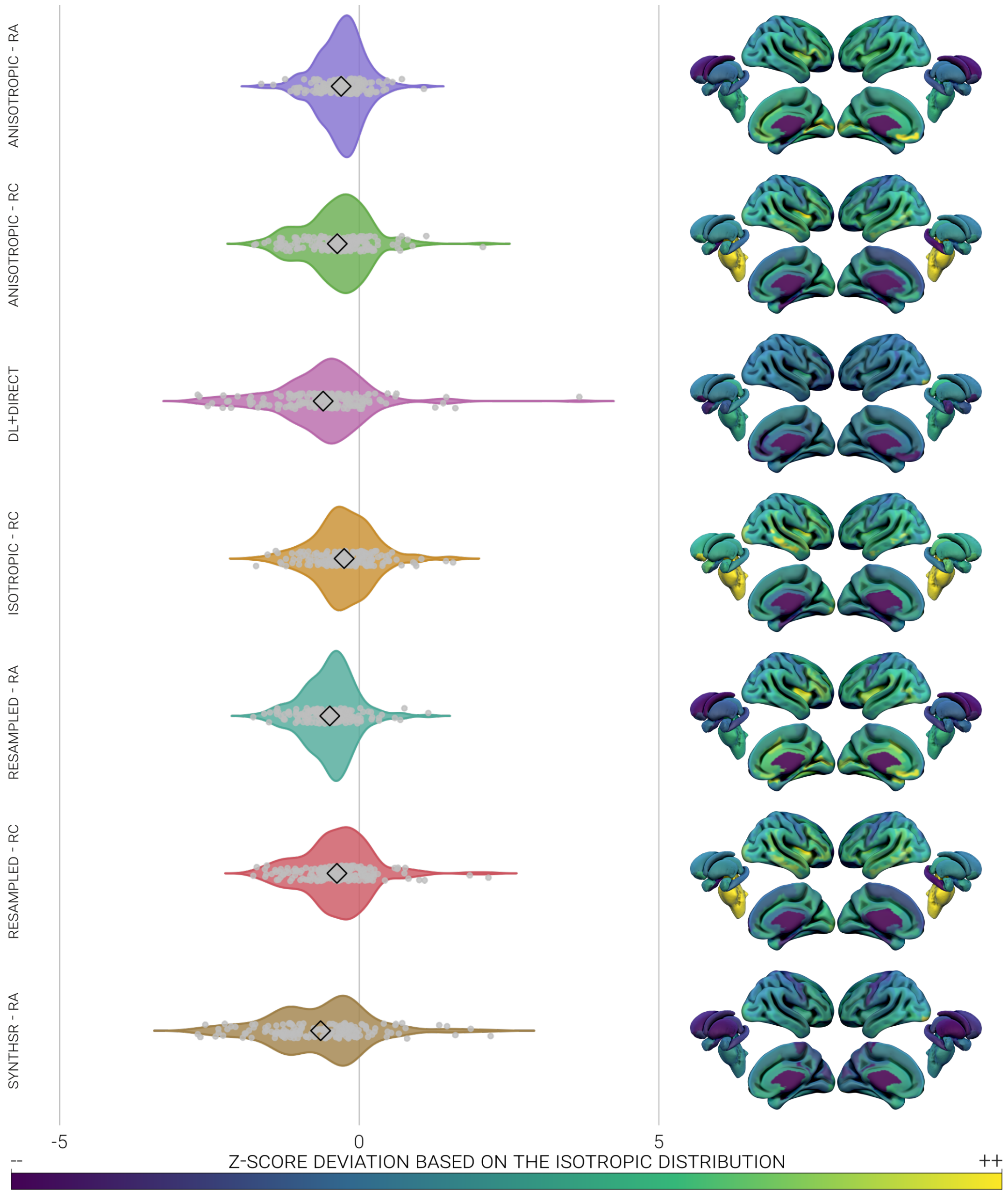

Figure s5. Heatmap and violin plot representations of the volume estimation variance (presented on the scale of -5 to +5 SD) of 174 parcellated regions created from isotropic scans and the various analogues. Regional volume estimates were first standardised to the distribution of the same region in the isotropic data. Regional data were averaged across all participants in each image type, to leave one datum per region per analogue. For heatmap creation, the values were then normalised to between 0 and 1 for each image type - these heatmaps therefore represent relative distributions of estimation bias, with the scale being bounded to the minimum and maximum z-scores for that image type. RA, `recon-all`; RC, `recon-all-clinical`

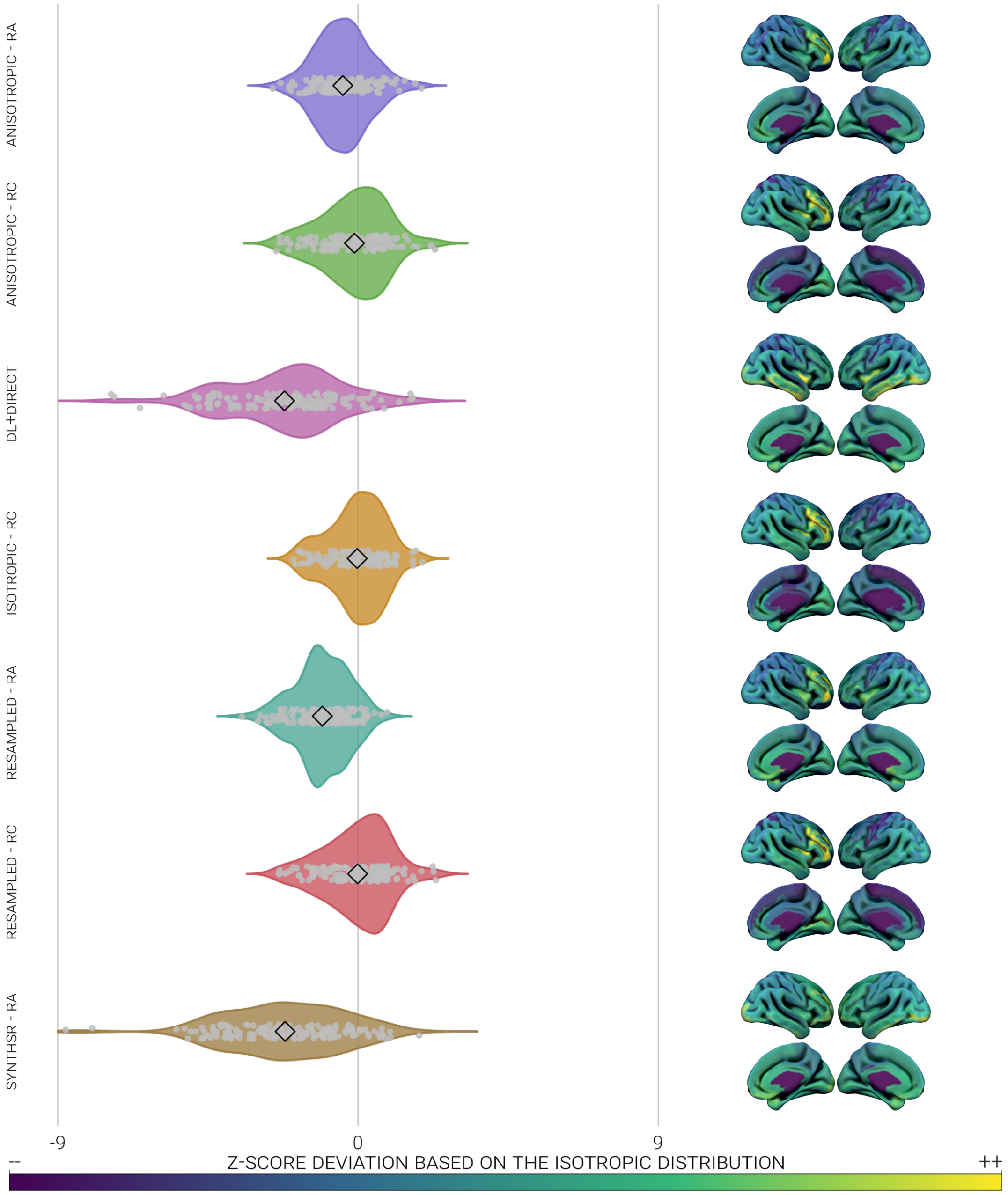

Figure s6. Heatmap and violin plot representations of the thickness estimation variance (presented on the scale of -9 to +9 SD) of 148 parcellated regions created from isotropic scans and the various analogues. Regional thickness estimates were first standardised to the distribution of the same region in the isotropic data. Regional data were averaged across all participants in each image type, to leave one datum per region per analogue. For heatmap creation, the values were then normalised to between 0 and 1 for each image type - these heatmaps therefore represent relative distributions of estimation bias, with the scale being bounded to the minimum and maximum z-scores for that image type. RA, `recon-all`; RC, `recon-all-clinical`

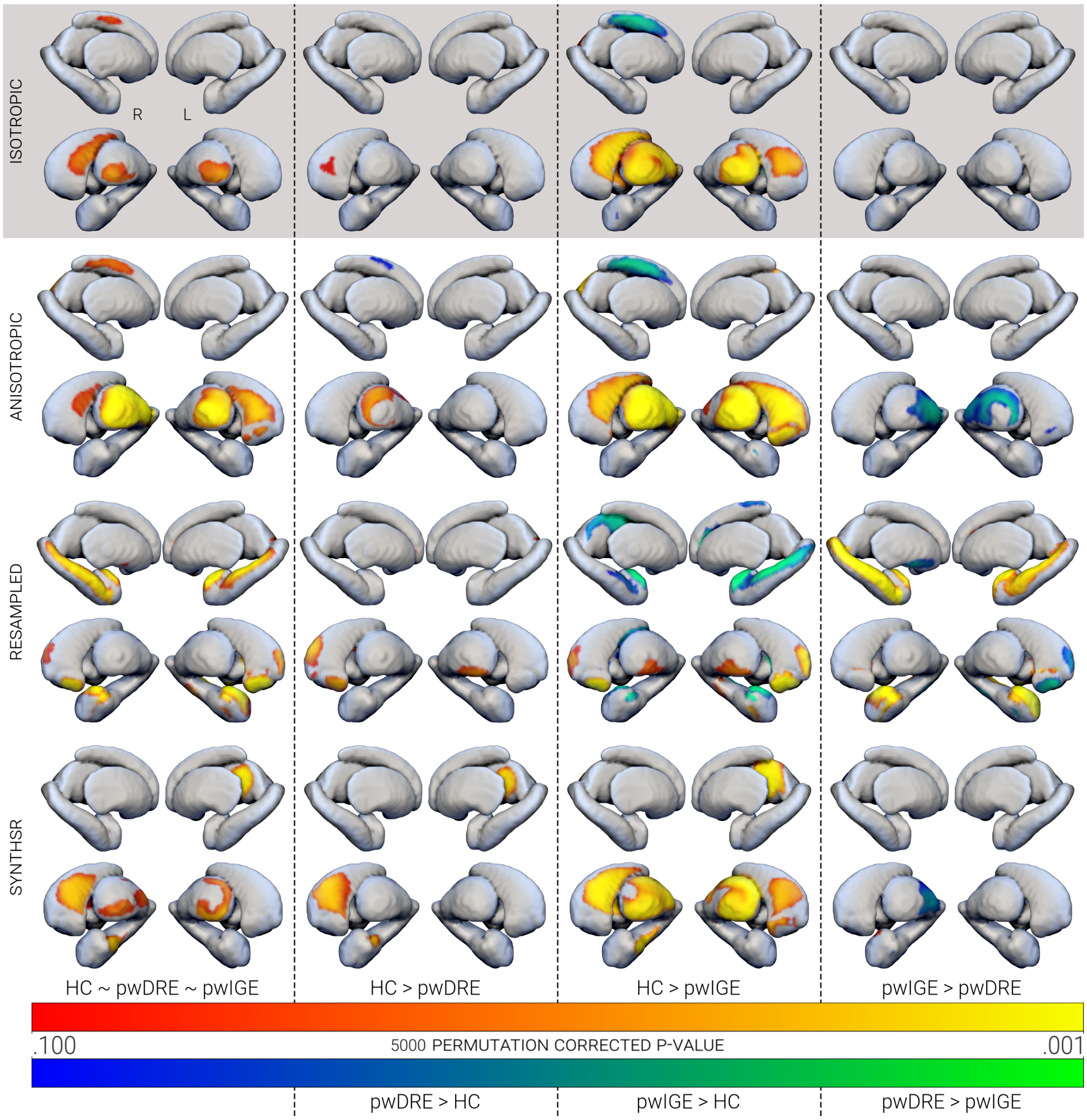

Figure s7. Clusters of subcortical surface shape deformations between people with drug-resistant IGE, people with drug-sensitive IGE, and healthy controls, observed across image types. Only clusters with *p corr* < .100 are shown, which were derived with one-sided permutation (*n-perm* = 5000) testing. The direction of the contrast is presented next to the relevant colourbar. HC, Healthy Controls; IGE, Idiopathic Generalised Epilepsy; pwDRIGE, people with drug-resistant IGE; pwDSIGE, people with drug-sensitive IGE

Supplementary Material S5

Supplementary Material S5.a.

R environment

- R (R Core Team, 2023)
- RStudio (RStudio Team, 2020)

Supplementary Material S5.b.

R packages

- car (Fox et al., 2024)
- forcats (Wickham & RStudio, 2023)
- ggplot2 (Wickham, 2016)
- ggpubr (Kassambara, 2025)
- MiscTools (Henningsen & Toomet, 2019)
- psych (Revelle, 2023)
- RNifti (Clayden et al., 2023)
- svglite (Wickham, Henry, et al., 2023)
- tidyr (Wickham, Vaughan, & Girlich, 2023)

Supplementary Material S5.c.

Visualisation software

- GIMP (The GIMP Development Team, 2019)
- Inkscape (Inkscape Project, 2020)
- MRICroGL (Rorden & Brett, 2000)
- Scribus (The Scribus Team, 2023)
- Surf Ice (Rorden, 2021)

Supplementary Material S5.d.

Image analysis software

- ANTs (Avants et al., 2009)
- FMRIB Software Library (Jenkinson et al., 2012)
- FreeSurfer (Fischl, 2012)
- Nibabel (Brett et al., 2023)
- DL+DiReCT (McKinley et al., 2021; Rebsamen et al., 2020, 2022)
- SynthSR (Billot et al., 2023; Iglesias et al., 2023)

References

Avants, B. B., Tustison, N., & Johnson, H. (2009). Advanced Normalization Tools (ANTS). *Insight J*, *2*(365), 1–35.

Billot, B., Greve, D. N., Puonti, O., Thielscher, A., Van Leemput, K., Fischl, B., Dalca, A. V., & Iglesias, J. E. (2023). SynthSeg: Segmentation of brain MRI scans of any contrast and resolution without retraining. *Medical Image Analysis*, *86*, 102789. https://doi.org/10.1016/j.media.2023.102789

Brett, M., Markiewicz, C. J., Hanke, M., Côté, M.-A., Cipollini, B., McCarthy, P., Jarecka, D., Cheng, C. P., Halchenko, Y. O., Cottaar, M., Larson, E., Ghosh, S., Wassermann, D., Gerhard, S., Lee, G. R., Baratz, Z., Wang, H.-T., Kastman, E., Kaczmarzyk, J., … freec84. (2023, April). Nipy/nibabel: 5.1.0. https://doi.org/10.5281/zenodo.7795644

Clayden, J., Cox, B., Jenkinson, M., Hall, M., Reynolds, R., Fissell, K., Gailly, J.-l., & Adler, M. (2023). RNifti: Fast R and C++ Access to NIfTI Images.

Fischl, B. (2012). FreeSurfer. *NeuroImage*, *62*(2), 774–781. https://doi.org/10.1016/j.neuroimage.2012.01.021

Fox, J., Weisberg, S., Price, B., Adler, D., Bates, D., Baud-Bovy, G., Bolker, B., Ellison, S., Firth, D., Friendly, M., Gorjanc, G., Graves, S., Heiberger, R., Krivitsky, P., Laboissiere, R., Maechler, M., Monette, G., Murdoch, D., Nilsson, H., … R-Core. (2024, September). Car: Companion to Applied Regression.

Henningsen, A., & Toomet, O. (2019). Misctools: Miscellaneous Tools and Utilities.

Iglesias, J. E., Billot, B., Balbastre, Y., Magdamo, C., Arnold, S. E., Das, S., Edlow, B. L., Alexander, D. C., Golland, P., & Fischl, B. (2023). SynthSR: A public AI tool to turn heterogeneous clinical brain scans into high-resolution T1-weighted images for 3D morphometry. *Science Advances*, *9*(5), eadd3607. https://doi.org/10.1126/sciadv.add3607

Inkscape Project. (2020, April). Inkscape.

Jenkinson, M., Beckmann, C. F., Behrens, T. E., Woolrich, M. W., & Smith, S. M. (2012). FSL. *NeuroImage*, *62*(2), 782–790. https://doi.org/10.1016/j.neuroimage.2011.09.015

Kassambara, A. (2025, June). Ggpubr: ’ggplot2’ Based Publication Ready Plots.

McKinley, R., Wepfer, R., Aschwanden, F., Grunder, L., Muri, R., Rummel, C., Verma, R., Weisstanner, C., Reyes, M., Salmen, A., Chan, A., Wagner, F., & Wiest, R. (2021). Simultaneous lesion and brain segmentation in multiple sclerosis using deep neural networks. *Scientific Reports*, *11*(1), 1087. https://doi.org/10.1038/s41598-020-79925-4

R Core Team. (2023). R: A Language and Environment for Statistical Computing.

Rebsamen, M., McKinley, R., Radojewski, P., Pistor, M., Friedli, C., Hoepner, R., Salmen, A., Chan, A., Reyes, M., Wagner, F., Wiest, R., & Rummel, C. (2022). Reliable brain morphometry from contrast-enhanced T1w-MRI in patients with multiple sclerosis. *Human Brain Mapping*, *44*(3), 970–979. https://doi.org/10.1002/hbm.26117

Rebsamen, M., Rummel, C., Reyes, M., Wiest, R., & McKinley, R. (2020). Direct cortical thickness estimation using deep learning-based anatomy segmentation and cortex parcellation. *Human Brain Mapping*, *41*(17), 4804–4814. https://doi.org/10.1002/hbm.25159

Revelle, W. (2023). Psych: Procedures for psychological, psychometric, and personality research.

Rorden, C. (2021). Surf Ice: A Simple Tool for Visualizing Connectome Networks, Tractography, and Statistical Maps.

Rorden, C., & Brett, M. (2000). Stereotaxic Display of Brain Lesions. *Behavioural Neurology*, *12*(4), 191–200. https://doi.org/10.1155/2000/421719

RStudio Team. (2020). RStudio: Integrated development environment for R.

The GIMP Development Team. (2019, June). GIMP.

The Scribus Team. (2023). Scribus: Open Source Desktop Publishing.

Wickham, H. (2016). Ggplot2: Elegant graphics for data analysis.

Wickham, H., Henry, L., Pedersen, T. L., Luciani, T. J., Decorde, M., & Lise, V. (2023). Svglite: An 'SVG' graphics device.

Wickham, H., & RStudio. (2023, January). Forcats: Tools for Working with Categorical Variables (Factors).

Wickham, H., Vaughan, D., & Girlich, M. (2023). Tidyr: Tidy messy data.
